# A machine learning approach to identifying suicide risk among text-based crisis counseling encounters

**DOI:** 10.3389/fpsyt.2023.1110527

**Published:** 2023-03-23

**Authors:** Meghan Broadbent, Mattia Medina Grespan, Katherine Axford, Xinyao Zhang, Vivek Srikumar, Brent Kious, Zac Imel

**Affiliations:** ^1^Educational Psychology, The University of Utah, Salt Lake City, UT, United States; ^2^Kahlert School of Computing, The University of Utah, Salt Lake City, UT, United States; ^3^Department of Psychiatry, The University of Utah, Salt Lake City, UT, United States

**Keywords:** machine learning, suicide, crisis text-line, text content, natural language processing

## Abstract

**Introduction:**

With the increasing utilization of text-based suicide crisis counseling, new means of identifying at risk clients must be explored. Natural language processing (NLP) holds promise for evaluating the content of crisis counseling; here we use a data-driven approach to evaluate NLP methods in identifying client suicide risk.

**Methods:**

De-identified crisis counseling data from a regional text-based crisis encounter and mobile tipline application were used to evaluate two modeling approaches in classifying client suicide risk levels. A manual evaluation of model errors and system behavior was conducted.

**Results:**

The neural model outperformed a term frequency-inverse document frequency (tf-idf) model in the false-negative rate. While 75% of the neural model’s false negative encounters had some discussion of suicidality, 62.5% saw a resolution of the client’s initial concerns. Similarly, the neural model detected signals of suicidality in 60.6% of false-positive encounters.

**Discussion:**

The neural model demonstrated greater sensitivity in the detection of client suicide risk. A manual assessment of errors and model performance reflected these same findings, detecting higher levels of risk in many of the false-positive encounters and lower levels of risk in many of the false negatives. NLP-based models can detect the suicide risk of text-based crisis encounters from the encounter’s content.

## 1. Introduction

Suicide and crisis hotlines can be an effective, inexpensive, and accessible resource for people in crisis in need of confidential support (1, 2). These interventions can help deescalate and reduce feelings of distress and hopelessness (2, 3) as well as decrease the likelihood of attempting suicide (4, 5). In the last decade, text-messaging has become a dominant form of communication generally and in mental health care, especially among youth who may prefer text-messaging as a more immediate, private, and familiar modality (2, 6–12). As such, text-based crisis counseling has begun to supplement phone-based conversations with tens of thousands of crisis messages sent each day (13). While the reach of text messaging is impressive, this volume of care places a tremendous burden on crisis systems to train and support counselors who are in short supply and at risk of burnout (14–19). Ensuring the consistent provision of high-quality crisis services is critical to their effectiveness (20, 21), and one such way to support this is through accurate and consistent evaluation of the level of risk in a given crisis conversation.

Current guidelines on crisis counseling and suicide risk assessment suggest a minimum of three questions to evaluate risk of suicide (20, 21). However, the consistency in following these guidelines in crisis services can vary dramatically, with some studies showing that crisis clients are often not asked about suicidal ideation (3, 22), and one study in particular finding no assessment of suicide risk in over half of all telephone crisis calls (23). However, it should be noted that counselors in crisis settings, especially text-based crisis settings, are often navigating these complex conversations with multiple clients simultaneously. While counselors, who often have relatively minimal training, are expected to report on risk after a conversation as a means of quality assurance, counselors are navigating many conflicting demands (e.g., responding to the next texter promptly and empathically vs. generating thorough documentation). Given broad concerns about the accuracy of documentation in medical records (24), it is possible they may overlook risk in a conversation or may simply forget to document risk in the midst of other competing tasks. It is imperative we explore new means of supporting counselors in the identification of at-risk clients.

Natural language processing (NLP) is one tool that holds significant promise for developing scalable methods for evaluating the content of crisis counseling (25). A subfield of artificial intelligence and linguistics, NLP methods enable a computerized approach to the learning, interpretation, processing, and analysis of human language in written or spoken form (26–28). Modern NLP methods rely on a family of machine learning models called neural networks that are trained to encode linguistic information from input text data for a specific task such as text classification, text summarization, and text translation (29–31). There have been notable efforts in recent years to deploy NLP methods in psychotherapy and mental health research (32, 33), with numerous studies showing potential success in identifying and predicting instances of suicidality across a variety of text-based sources including clinical records, discharge notes, patient-therapist dialogues, and social media posts (34–40). While this evidence suggests risk in text-based mental health counseling can be estimated using NLP-methods, research evaluating risk from clinical dialogues has focused on general asynchronous counseling environments where the risk of suicide is lower than in crisis counseling (33). Most recently, one study used domain knowledge to encode the content of the conversations for risk assessment (41). This work is promising but relies on the necessarily incomplete theoretical frameworks of experts, rather than a data-driven approach to learning associations between text and suicide risk, likely reducing generalizability and performance. There is no published application of modern transformer-based language models to risk identification in crisis counseling.

In this study, we build upon recent advancements in NLP using a data-driven approach to train and test NLP-methods on naturalistic crisis counseling data in identifying the presence of suicide risk. Specifically, we present a modern transformer-based neural architecture powered by state-of-the-art Robustly Optimized BERT Pre-training Approach (RoBERTa) embeddings trained over large, labeled crisis conversations from a regional crisis counseling app (42). It is possible for neural network models to learn spurious correlations based on artifacts of data collected (43, 44). Accordingly, we also conducted a thorough analysis of model errors and system behavior including a manual evaluation of encounters associated with model errors and cumulative risk throughout a crisis counseling dialogue.

## 2. Materials and methods

### 2.1. Data

This retrospective study utilized de-identified data from 5,992 crisis counseling encounters (totaling 273,804 messages) collected from SafeUT, a regional text-based crisis encounter and mobile tip line app (see Table 1 for a data summary). The SafeUT counselors are licensed or license-eligible clinical social workers with a background in crisis counseling. SafeUT counselors receive additional training in suicide risk assessment and safety planning. The study sample included crisis encounters from clients of any age located in Utah, Idaho, and Nevada who utilized the service between June 2020–April 2021. Mobile tips, a system for notifying schools and educators about potentially at-risk student peers, were excluded from the study sample. Institutional Review Board (IRB) approval was obtained for this study. SafeUT does not systematically collect potentially identifiable information and text messages were scrubbed of incidental identifying information prior to analysis.

**TABLE 1:** Data summary of crisis counseling encounters.

Encounter measure	Minimum	Median	Mean (SD)	Maximum
Duration (minutes)	0.014	0.950	1.106 (0.776)	9.605
Number of counselors	1	1	1.404 (0.574)	5
Counselor messages	1	17	20.973 (14.568)	159
Client messages	1	20	24.840 (19.427)	287

### 2.2. Measure

Dispositions of each crisis counseling encounter, labeled by the SafeUT counselors, were used to measure the level of client risk. Counselor-generated dispositions cover a range of topics discussed, services provided, type and level of action needed, client perceptions of crisis counseling interaction, as well as the degree of client suicide risk perceived by the counselor. For the latter, counselors are asked to follow the Suicide Risk Assessment Standards of the National Suicide Prevention Lifeline (45). This guideline, recommends crisis workers to a minimum number of suicide status prompt questions (see Supplementary Appendix I for more details). Importantly, crisis workers are instructed to mark the degree of risk in a way that reflects the whole encounter in aggregate.

Counselors assign suicide risk labels (i.e., dispositions) to each crisis counseling encounter, with options ranging from low-risk, moderate-risk, high-risk, and emergency referral (mobile crisis outreach team response, active rescue by law enforcement or paramedics, school contact). Counselors evaluate suicide risk level, based on clients’ self-report, using their clinical judgment with respect to the intensity of reported suicidal ideation and other clinical risk factors (such as access to lethal means) that are endorsed by the client. Overall, 85.3% of all crisis counseling encounters were categorized as “lower risk,” followed by 9.25% as moderate-risk encounters, 3.47% as high-risk encounters, and 1.95% as emergency-referral encounters. For the purposes of this study, we collapsed these ratings into a binary label classifying risk as either “lower risk” or “higher risk” to form more even groups based on sample size and to allow for a logistic regression analysis (where “higher risk” included all other categories except low-risk). Overall, 85.3% of all crisis counseling encounters were categorized as “lower risk,” followed by 9.25% as moderate-risk encounters, 3.47% as high-risk encounters, and 1.95% as emergency-referral encounters (resulting in 14.67% of all encounters categorized as having “higher risk;” Table 2).

**TABLE 2:** Categories of risk.

Category		*n*	%
Lower risk		5,113	85.3
Higher risk*	Moderate risk	554	9.25
	High risk	208	3.47
	Emergency referral	117	1.95

*n = 879 (14.6% of all crisis counseling encounters).

### 2.3. Model training and analysis

Two modeling approaches were evaluated for the classification of risk level in crisis encounters, a neural network model and a term frequency-inverse document frequency (tf-idf) weighted logistic regression model for a baseline comparison. Existing counseling-generated risk dispositions were used to train both models in classifying the level of risk for each crisis counseling encounter. Both models were evaluated using the receiver operating characteristic area under the curve (ROC AUC). The higher the AUC score the better the model classification, with an AUC of 1 suggesting a perfect (but likely over-fitted) classifier, an AUC of 0.5 suggesting a random-chance classifier, and an AUC of 0.8 or higher suggesting a good classifier (46). Other evaluation measures included sensitivity, proportion of higher risk encounters correctly classified by the model; specificity, proportion of lower risk encounters correctly classified by the model; precision, proportion of correct higher risk predictions of the model; false-negative rate (1-sensitivity), and false-positive rate (1-specificity).

Both the neural network model and tf-idf model received a crisis counseling encounter as input and output a probability distribution for “lower risk” vs. “higher risk.” An 80/20 train-test split was used, with model training on 80% of the data and two hold-out (test) sets, each corresponding to 10% of the remaining data, for development and testing, respectively. This partition was done maintaining the same distribution of the labels across the three datasets.

### 2.4. Neural network

The neural network model utilized a machine learning transformer architecture. Transformers are a family of neural networks designed to process sequential data using self-attention, a mechanism allowing the network to extract and use information from arbitrarily large input contexts efficiently (47). The initial component in the transformer architecture (i.e., encoder) is particularly useful in natural language processing as it takes a string of text (sequence of words) and returns a sequence of numerical representations of the input corresponding to each word in the input text (47). These numerical representations (i.e., word embeddings) contain the semantic and grammatical meaning learned from context through the transformer’s self-attention process (47). Current state-of-the-art approaches in many NLP-tasks are based on RoBERTa embeddings, which are contextualized word embeddings obtained from stacking multiple transformer encoder blocks or layers pre-trained on large corpora of text (42, 48).

**FIGURE 1:**
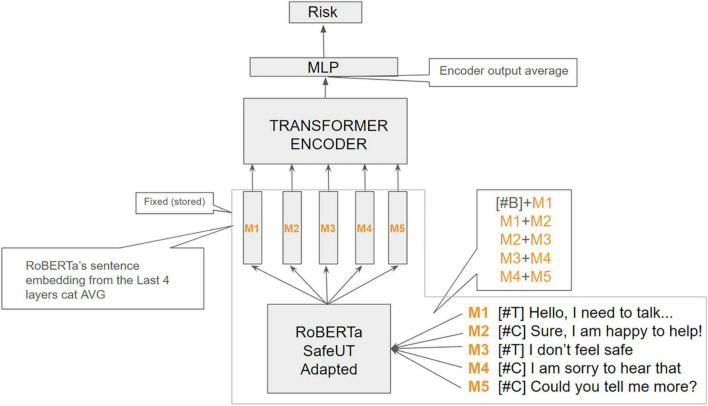
Architecture of the neural network model using an example of five messages. B, beginning token; C, client, M, message; MLP, Multiple-layer perception; T, therapist. Example using a 5-message encounter input. Special tokens (#T) and (#C) are prepared to each client and counselor message, respectively, to inform the model about the originator of each message along the conversation. We preprocessed each encounter by obtaining the aggregated RoBERTa sentence embeddings of every pair of consecutive message-spans in the encounter. Each obtained sentence embedding sequence is passed through a transformer-encoder block, whose output is aggregated to obtain an encounter embedding representation. The encounter vector is then fed to a final neural linear layer to obtain the binary confidence estimates for classification.

In this study, RoBERTa word embeddings were aggregated into a single sentence embedding for each message in each crisis counseling encounter.^1^ Importantly, to adapt the original general-purpose RoBERTa embeddings to the domain of crisis counseling (49), we continued pretraining the model using 120,000 encounters from the SafeUT app (almost 2.5 million messages). The originator of each message (client or counselor) was prepended to each message in an encounter, whereby the concatenation of each pair of consecutive messages in an encounter was provided as inputs to the language model (e.g., back-to-back messages were provided as a single message input). To obtain the embedding representation of an encounter, we averaged the output of 6 transformer encoder layers. Lastly, the encounter embedding was passed through a linear neural layer (a neural network with just one layer of nodes) for binary classification [Figure 1; (47)]. To measure the stability of the model, the training process was repeated and averaged across five different random seeds. We used the model with the best AUC performance in the development set (out of the five seeds) for error and system behavior analysis in later sections.

### 2.5. Tf-logistic regression

In this comparison approach, we vectorized the data using a term frequency-inverse document frequency statistic (tf-idf), combining unigrams (individual words) and bi-grams (pairs of consecutive words) from the messages on the sessions. A binary logistic regression classifier was trained on the vectorized data until convergence was achieved.

### 2.6. Error assessment

A manual evaluation of model errors was conducted to assess features of crisis counseling encounters falsely categorized as “higher risk” or “lower risk” by the neural model to better understand the behavior of the model and its prediction of risk. A team of four human reviewers assessed each encounter erroneously categorized by the neural model for indicators of suicidality, non-suicidal self-injury, abuse, emergency service triage, mobile tips, social service involvement, discussion of therapy services, and client drop-off (i.e., when the client stops responding to the encounter). The degree of resolution of the client’s complaint was also evaluated, with resolution indicating a near total reduction of a client’s risk or distress and/or de-escalation of client crisis at encounter end. Partial resolution similarly indicates some reduction of client distress with moderate to low client risk at end of the encounter. No resolution indicates minimal to no reduction of client risk or distress with the client remaining at risk at end of the encounter. Lastly, the team of reviewers made a final determination of whether the encounter should be labeled higher risk or lower risk based on the context and indicators of the encounter.

Moreover, the dynamics of the risk probability continuum within a crisis conversation were evaluated to better understand the cumulative signal of risk captured by the neural model as counselor-client dialogue develops. Three crisis counseling encounters were selected at random to illustrate the neural model’s performance in predicting actual higher risk (true positives), falsely predicting higher risk (false positives), and falsely predicting lower risk (false negatives). True negative encounters were excluded from this illustration as these were mostly flat lines indicating minimal to no signal of risk throughout a counselor-client dialogue. This secondary evaluation of model errors allowed for a deeper inspection of model behavior and content that drove the model predictions.

## 3. Results

**FIGURE 2:**
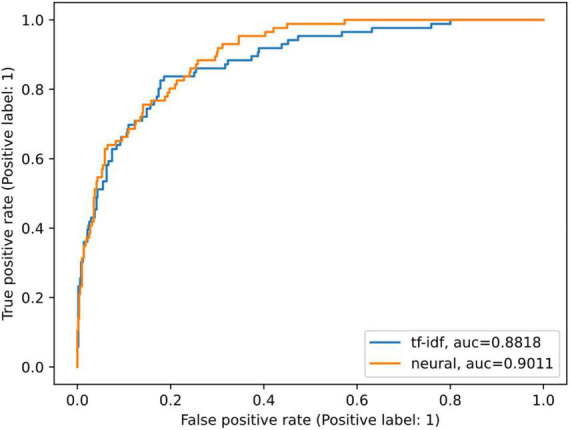
Comparison of model ROC AUCs. AUC, area under the curve; ROC, receiver operating characteristic. ROC curves of the tf-idf weighted logistic regression model (blue) and the neural model with the best performance in the development set out of the five runs (orange).

The neural model achieved an average AUC of 90.37% (Table 3). Precision, specificity, sensitivity, and other model metrics are reported in Table 3. In comparison with the neural model, the tf-idf model demonstrated a slight decrease in performance with an AUC of 88.17 (Figure 2 and Table 3).

**TABLE 3:** Model performance.

	Neural model (SD)	Tf-idf model
ROC AUC	90.37 (0.18)	88.18
Specificity	92.89 (0.46)	97.84
Sensitivity	62.02 (0.54)	39.52
Precision	59.52 (1.80)	75.56
FNR	37.98 (0.56)	60.47
FPR	7.11 (0.46)	2.16

FNR, false negative rate; FPR, false positive rate; SD, standard deviation. Average test performance across training with five different random seeds is shown in the neural model column.

Overall, the ROC curves and AUC scores of the two models are similar. Yet there are important differences. The false-negative rate of the neural model was relatively high at 37.98%, but it represents a 22.49-percentage point improvement in classifying risk compared to the tf-idf model at a 60.47% false-negative rate (Table 3). Similarly, the neural model had nearly double the sensitivity compared to the tf-idf model (62.02 vs. 39.52%, respectively; Table 3). The false-positive rate was low for both models, with the neural model slightly higher at 7.11%, highlighting the increased sensitivity of the neural model compared to the tf-idf model. Lastly, the tf-idf model demonstrated a higher positive predictive value and slightly higher specificity compared to the neural model (Table 3); highlighting the tf-idf model’s tendency to classify crisis counseling encounters as a lower risk compared to the neural model.

### 3.1. Manual assessment of errors

Based on counselor dispositions, the neural model incorrectly categorized a total of 32 encounters as lower risk (false negatives–i.e., the model labeled the encounter as lower risk when the human counselor had rated it as having significant risk) and 33 encounters as higher risk (false positives–i.e., the model predicted significant risk in the encounter when the human counselor had rated it as lower risk); Table 4; see Supplementary Appendix II for Tables 5, 6 detailing *ad hoc* human evaluation.

**TABLE 4:** Summary of manual error assessment.

Measure*		False negatives *N* = 32 encounters *n* (%)	False positives *N* = 33 encounters *n* (%)
Higher risk^a^		17 (53.1)	13 (39.4)
Suicidality		24 (75.0)	20 (60.6)
NSSI^b^		10 (31.3)	9 (27.3)
Abuse		6 (18.8)	11 (33.3)
Emergency triage^c^		6 (18.8)	12 (36.4)
Mobile tips		10 (31.3)	4 (12.1)
Social services		6 (18.8)	3 (9.1)
External therapy services		15 (46.9)	16 (48.5)
Client drop off^d^		11 (34.4)	14 (42.4)
Complaint resolution^e^	Yes Partial No	20 (62.5) 4 (12.5) 8 (25.0)	14 (42.4) 11 (33.3) 8 (24.2)

^a^Higher risk determination based on team assessment of encounters.

^b^Non-suicidal self-injury (NSSI) includes self-harm without intent to die, such as cutting.

^c^Emergency triage includes triaging to emergency responders, hospital emergency rooms, and mobile crisis outreach teams.

^d^Client drop-off indicates the client stopped responding to the counselor.

^e^Resolve indicates a reduction in client risk or distress and/or de-escalation of a client crisis.

*See Supplementary Appendix II for more details on encounter summaries and indicators.

A manual assessment of each falsely categorized encounter revealed that of the 32 encounters where the model rated risk low (when a human therapist had rated it higher risk), 46.9% were considered lower risk by the team assessment (Table 4). Moreover, the majority of these false-negative encounters also had no discussion of other concerning topics such as non-suicidal self-injury, abuse, or involvement of first responders or crisis services (68.7, 81.2, and 81.2%, respectively). While a 75% majority of false-negative encounters had some discussion of suicidality, 62.5% also saw a resolution of the client’s initial concerns and reasons for using the service, suggesting some potential appropriateness in the neural model’s assessment. On the other hand, only 40% of the encounters categorized as higher risk by the model but lower risk by the counselor (i.e., false positives) were determined to be lower risk by the team of reviewers, while 60.6% included discussions of suicidality. A substantial minority of encounters also included concerns with non-suicidal self-injury, abuse, or involvement of first responders or crisis services (27.3, 33.3, and 36.4%, respectively). Furthermore, the majority of the encounters saw only partial or no resolution of the client’s concerns using the service (Table 4).

One potential interpretation of these findings is that the models learned appropriate indicators of risk, making them robust to the inherent inconsistency noise of the human counselor labeling. As noted in the introduction, crisis counselors are responding to multiple high-stress situations and their ratings may not be without error. Furthermore, counselors have access to historical information from clients, such as prior utilization of the SafeUT app, that also may affect the risk assessment.

### 3.2. Dialogue risk curves

**FIGURE 3:**
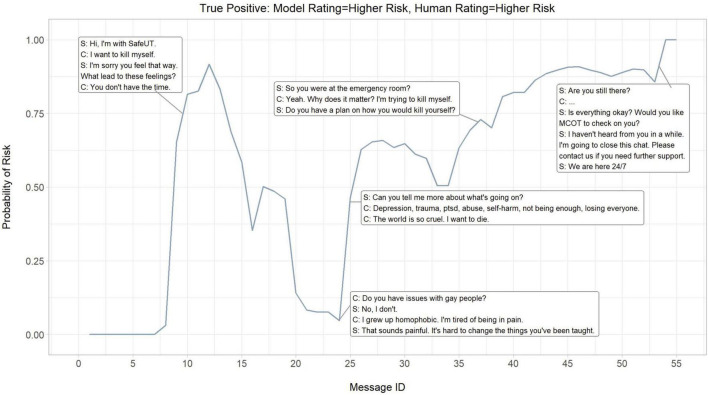
Dialogue risk curves: Model rating = higher risk, Human rating = higher risk (True positive). S, SafeUT counselor; C, SafeUT client; MCOT, Mobile Crisis Outreach Team. Crisis counseling encounter has been fictionalized to maintain confidentiality.

**FIGURE 4:**
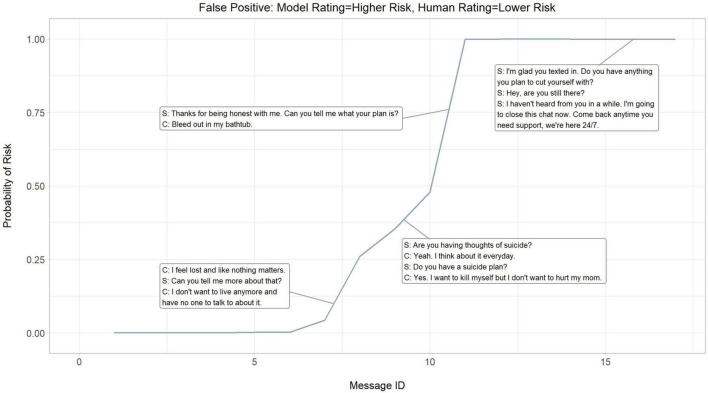
Dialogue risk curves: Model rating = higher risk, Human rating = higher risk (False positive). S, SafeUT counselor; C, SafeUT client. Crisis counseling encounter has been fictionalized to maintain confidentiality.

**FIGURE 5:**
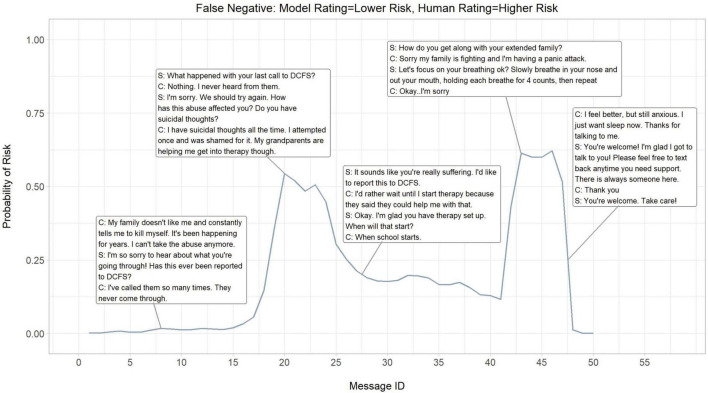
Dialogue risk curves: Model rating = lower risk, Human rating = lower risk (False negative). S, SafeUT counselor; C, SafeUT client; DCFS, Division of child and Family Services. Crisis counseling encounter has been fictionalized to maintain confidentiality.

To better understand the neural model’s performance, a cumulative probability of higher risk of each message and its contribution to an encounter was evaluated. The probability of higher risk throughout an encounter was visualized to demonstrate the continual dynamic of risk assessed by the neural model and where the neural model picks up on signals of risk. Three crisis counseling encounters were selected at random to illustrate the neural model’s performance in predicting actual higher risk (true positives; Figure 3), falsely predicting higher risk (false positives; Figure 4), and falsely predicting lower risk (false negatives; Figure 5).

Overall, the neural model appears to appropriately detect and predict both logical signals of risk and fluctuations in the degree of risk throughout a crisis counseling encounter. In the example where the neural model accurately predicts higher risk, a sudden increase in predicted higher risk is seen when a client states “I want to kill myself” (Figure 3). Moreover, the neural model seems to capture the higher risk associated with a distressed client who suddenly stops responding to the counselor. Interestingly, the neural model demonstrates an ability to capture the fluctuations in risk even in cases where it inaccurately predicts the level of risk (Figures 3, 4). While the counselor disposition in Figure 4 did not indicate higher risk, the neural model picks up on signals of higher risk when the client confirms suicidality with intent and similarly stops responding to the counselor; this might suggest that the neural model is able to aid a counselor in detecting higher risk. Similar to the examples in Figures 3, 4, the neural model demonstrates a sensible prediction of risk for the false-negative encounter in Figure 5 (where the counselor indicated higher risk and the neural model assigned lower risk). Overall, the neural model positively reflects a higher risk throughout this encounter when suicidality and active distress are present (Figure 5). However, the neural model seems to detect the reduced risk associated with casual conversation and the overall diffusion of the client’s distress at the end of the encounter.

## 4. Discussion

The current study illustrates the development and predictive utility of an NLP-algorithm to detect and classify the level of client suicide risk in a text-based crisis counseling environment. We examined a large sample of anonymous clients engaged in crisis counseling through the SafeUT platform, analyzing the content of crisis counseling text messages with SafeUT counselors.

Overall, the neural model yielded an excellent ROC AUC score of 0.9037. While the false positive and negative rates were higher than ideal (7.11 and 37.98%, respectively), a manual assessment of errors and evaluation of model performance throughout an encounter revealed the neural model was able to detect legitimate signals of higher risk in many of the false-positive encounters as well as lower levels of risk in many of the false negatives. This suggests that the neural model detected signals of risk even when learning from imperfect data. These findings further add support to the use of NLP methods as a potentially effective tool for aiding counselors in evaluating client risk in a text-based crisis counseling environment. This has important practice implications, not only for improving crisis counseling services but also to inform training and best practices for mental health counselors providing those services. In particular, eventual applications of systems like the one evaluated in this article could be used in parallel–not as a replacement–to provider assessment of risk. System detection of valid indicators of risk not identified by providers could provide an important stopgap in documenting key clinical processes when busy providers might be distracted by the next important clinical need.

Clear distinctions exist between our work and more recent efforts in automatic suicide risk detection in mental health counseling. Other studies have relied primarily on telemedicine psychotherapy dyads data including non-crisis interventions, making the base rate of suicide-related content dramatically different from the one found in an exclusive crisis counseling service. The work that pioneered this line of research reports models trained only with client messages from asynchronous encounters, hindering their system from learning crucial aspects of the task such as real-time risk dynamics of counseling, and the effect of counselor messages within the conversation (33). A more recent paper relies on data obtained from a similar text-based counseling platform, encoding entire encounters including counselor and client messages, as we do in our study (41). The authors introduce an interesting model with a knowledge-aware encoding layer obtained from a knowledge graph constructed by mental health experts demonstrating its efficacy through ablation studies. A downside of this kind of handcrafted database approach is the quality analysis, namely how to evaluate the completeness and correctness of the graph (50). In contrast, we propose a purely data-driven system where any existing relationships between concept words is extracted directly from the semantic and syntactic information present in the data. We argue that our approach is more robust and flexible when translated to other clinical fields, as it would only need the new dataset (the more data the better) without the need to rebuild a knowledge database that could depend not only on the specific domain expertise, but also on important aspects like cultural background of the study, data coming from a different language, or domain knowledge availability.

A contribution we consider unique in this article is that we provide detailed descriptive analyses of model results to evaluate the disagreements between the model and counselors who originally labeled the interactions. We had four clinical scientists read and relabel each encounter that had a predicted label different from the original counselor-generated label. From these experiments, we observed that for a significant number of encounters, the experts agreed with the neural model. This may indicate that our model is generalizing beyond the noise usually present in the labeling of clinical assessment datasets like this one. We extended our error analysis by creating dialog risk plots for such encounters, observing that our model captures risk in sync with the dynamics of the conversation. Although more analysis is needed, these results suggest that it is possible to obtain message-level supervision from encounter-level risk disposition labeling. We consider this observation a promising opportunity for future work.

### 4.1. Limitations

While this study examined suicide risk with reasonably high performance, it relied on a single crisis counseling encounter source for data. Moreover, the true risk of suicidality could not be reliably determined and instead relied on counselor-provided dispositions. These dispositions, used for model training, depend on accurate labeling from the counselors. Similarly, counselors may have access to historical information, such as prior utilization of the SafeUT app. It is possible that counselors do not adhere to the same standards for disposition selection or consideration of historical client information, potentially biasing model training results. This is further suggested by the manual assessment of errors and evaluation of dialogue arcs, with some “lower risk” crisis counseling encounters (deemed “lower risk” by the counselor dispositions and classified as having “higher risk” by the neural model) found to contain suicide-related content or risk-associated discourse (e.g., active self-harm, abuse, and requests for emergency responders). As such, future research should utilize human coding to evaluate and establish a baseline for suicidality (both client expression and counselor assessment). It is important to point out that in spite of the advances brought by modern NLP methods to the mental health community, further studies need to be done to assess the robustness of these systems (51–53). Lastly, client demographic data was not available for this study. Large language models have been shown to underperform when utilized by populations these models were not trained on (54–57). As such, it is possible these study findings may not generalize to populations whose racial, ethnic, or cultural demographics differ from the population in this study.

## 5. Conclusion

We observed that NLP-based models are capable of detecting suicide risk at the conversation level on text-based crisis encounters, suggesting important practice implications. Our results show outstanding AUC score performance, and remarkable precision and recall metrics by a modern neural transformer-based architecture. Manual analysis indicates that these models can learn appropriate indicators of risk making them robust to the inherent noise of the labels in real-time crisis encounters services. Furthermore, the dialog risk curves are a novel demonstration of how risk prediction fluctuates at the message level, capturing the dynamics of risk throughout the different stages of real crisis counseling conversations.

## Data availability statement

The datasets presented in this article are not readily available. Due to privacy and ethical concerns, the data cannot be made public. Requests to access the datasets should be directed to KA, kate.axford@utah.edu.

## Ethics statement

The studies involving human participants were reviewed and approved by the University of Utah Institutional Review Board. Written informed consent from the participants’ legal guardian/next of kin was not required to participate in this study in accordance with the national legislation and the institutional requirements.

## Author contributions

MB and MM contributed to the data preparation and analysis. MB, MM, and KA took the lead in the writing and preparation of the manuscript. MM, XZ, and VS designed the model. VS, BK, and ZI provided the feedback and insight that facilitated the analysis and manuscript. All authors contributed to the overall end product, contributed to the article, and approved the submitted version.

## Footnotes

1 The size of the encounters and hardware limitations prevented us from fine-tuning the RoBERTa model.
