# Supplementary material for: A machine learning approach to identifying suicide risk among text-based crisis counseling encounters
Source: Front Psychiatry. 2023 Mar 23;14:1110527. doi: 10.3389/fpsyt.2023.1110527 (PMC10076638; doi:10.3389/fpsyt.2023.1110527)
Supplement: Supplementary file 1 [file Data_Sheet_1.docx]

## APPENDIX I


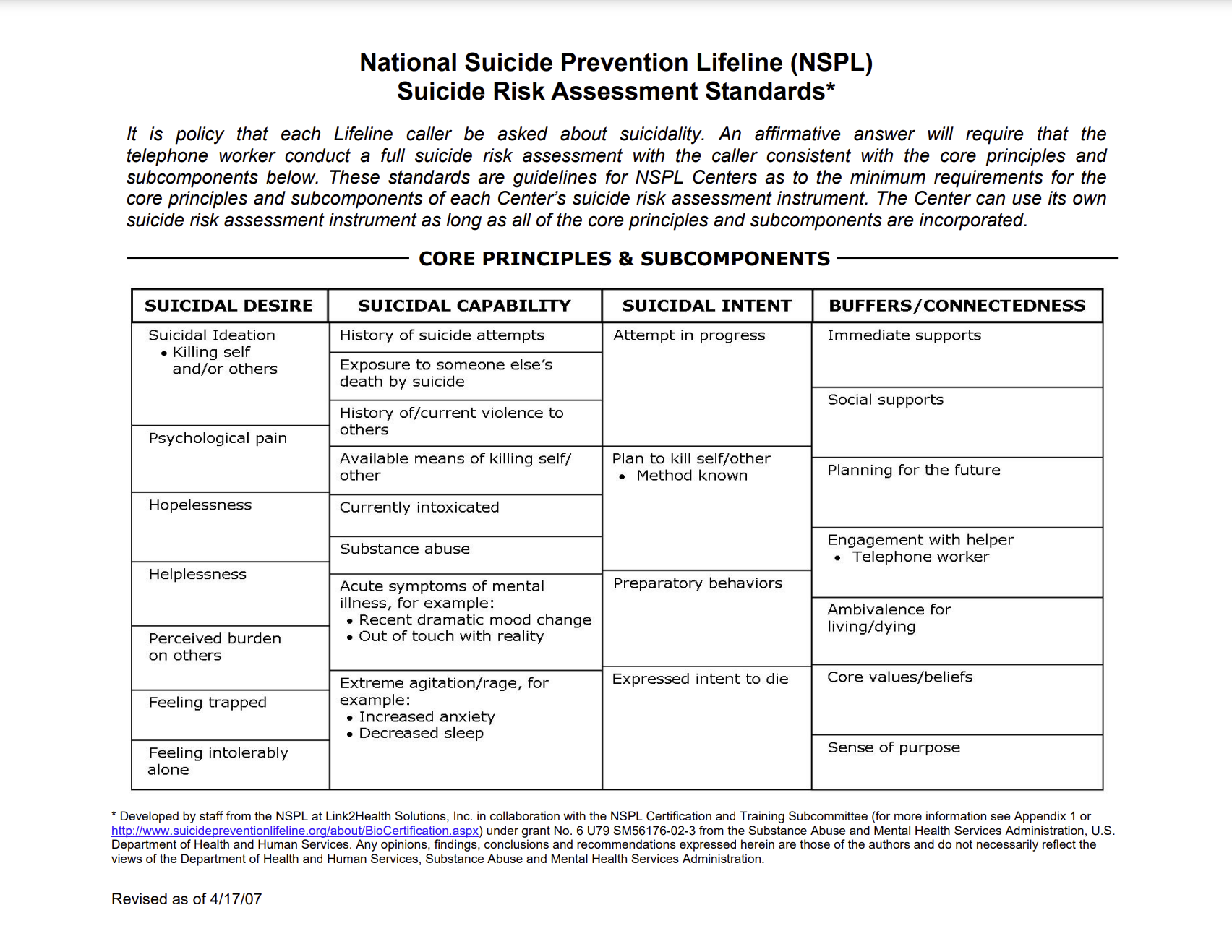


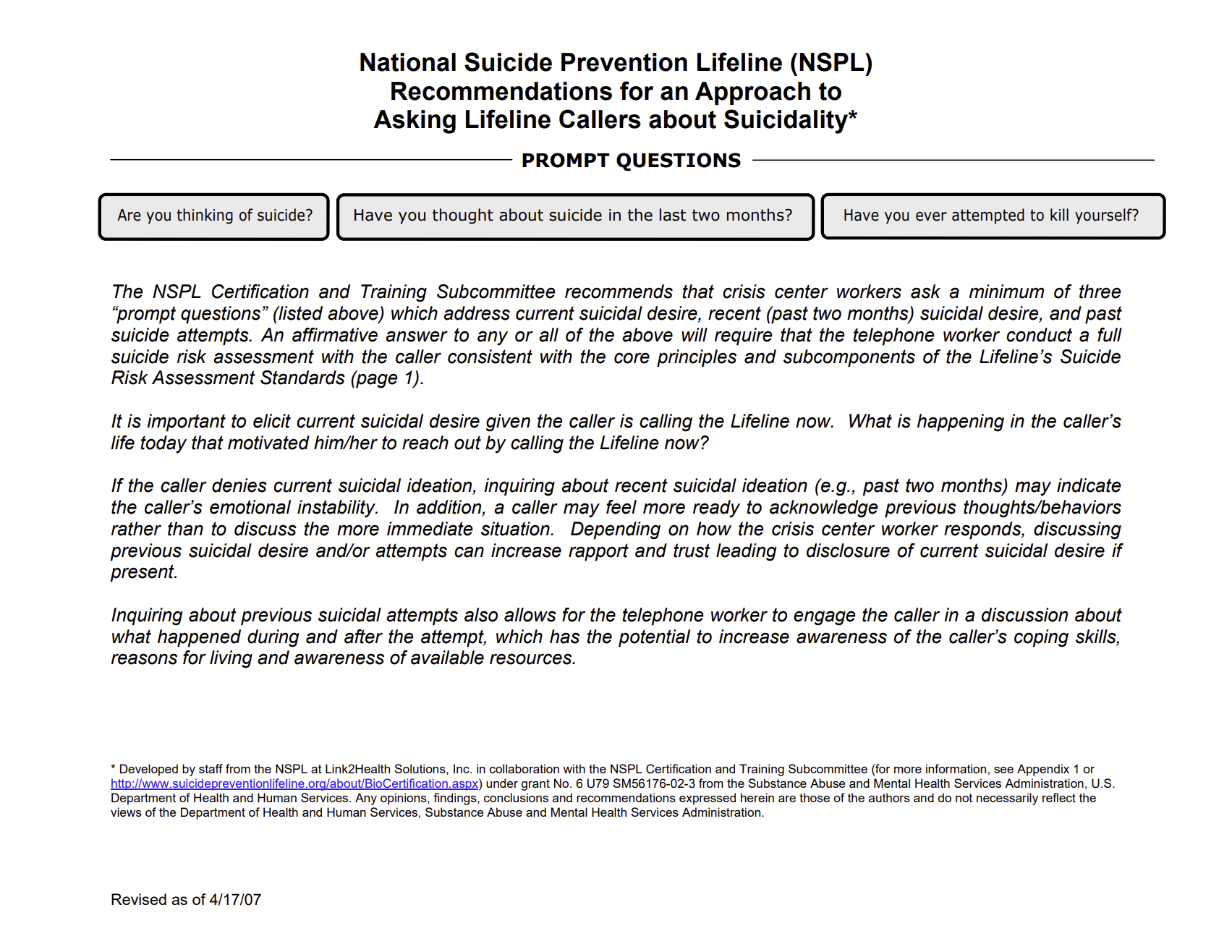


## APPENDIX II

Table 5. Model Rating=Low, Human Rating=Risk (False Negative)

|  |  | Post-hoc Human Rating | | | | | | | | | |
| --- | --- | --- | --- | --- | --- | --- | --- | --- | --- | --- | --- |
| ID | Encounter Summary | Suicidality | NSSI^a^ | Abuse | Emergency Triage^b^ | Mobile Tips | Social Services | External Therapy Services | Client Drop Off^c^ | Resolve^d^ | Risk^e^ |
| 1 | Parent seeking help for child in distress and at risk of suicide. | ✖ |  |  | ✖ |  |  | ✖ |  | Yes | ✖ |
| 2 | Client struggling with suicidal ideation; difficulty with accessing resources. | ✖ |  |  | ✖ |  |  | ✖ |  | Yes | ✖ |
| 3 | Parent seeking help for child’s friend in abusive, self-harming relationship. |  | ✖ | ✖ |  | ✖ | ✖ |  |  | Yes |  |
| 4 | Client struggling with abusive family. |  |  | ✖ |  |  | ✖ |  | ✖ | Yes |  |
| 5 | Possible client suicide attempt; struggling with ideation. | ✖ |  |  | ✖ |  |  | ✖ | ✖ | No | ✖ |
| 6 | Client struggling with active self-harm. |  | ✖ |  |  |  |  |  |  | Yes |  |
| 7 | Suicidal client struggling with abusive family and barriers to resources. | ✖ | ✖ | ✖ |  |  | ✖ | ✖ |  | Yes | ✖ |
| 8 | Client feeling unmotivated; struggling with poor habits. |  |  |  |  |  |  |  |  | Yes |  |
| 9 | Client struggling with breakup, suicidality, and emotional triggers. | ✖ | ✖ |  |  |  |  | ✖ |  | Yes |  |
| 10 | Client struggling with abusive family and challenges with social services. |  |  | ✖ |  | ✖ | ✖ | ✖ |  | Yes |  |
| 11 | Peer seeking help for suicidal friend experiencing familial abuse. | ✖ |  | ✖ | ✖ | ✖ | ✖ |  |  | Yes |  |
| 12 | Client struggling with suicidality, anger, and depression. | ✖ |  |  |  |  |  | ✖ |  | No | ✖ |
| 13 | Suicidal client struggling with family issues. | ✖ |  |  |  |  |  |  | ✖ | No | ✖ |
| 14 | Suicidal client struggling with loneliness and family issues. | ✖ |  |  |  |  |  | ✖ |  | Partial | ✖ |
| 15 | Parent seeking help for child’s ex threatening suicide. | ✖ |  |  |  | ✖ |  |  | ✖ | Yes |  |
| 16 | Client struggling with depression, relationship issues, and emotional triggers. | ✖ | ✖ |  |  |  |  |  | ✖ | No | ✖ |
| 17 | Suicidal client struggling with depression and poverty. | ✖ | ✖ |  |  |  |  |  |  | Yes |  |
| 18 | Peer seeking help for a suicidal friend. | ✖ |  |  |  | ✖ |  |  |  | Yes | ✖ |
| 19 | Client struggling with suicidal ideation and academic stressors. | ✖ |  |  |  |  |  | ✖ | ✖ | Partial | ✖ |
| 20 | Peer seeking help for a suicidal friend. | ✖ |  |  |  | ✖ |  |  | ✖ | No | ✖ |
| 21 | Client struggling with suicidal ideation and contacting their therapist. | ✖ | ✖ |  | ✖ |  |  | ✖ |  | Partial | ✖ |
| 22 | Peer seeking help for friend struggling with severe depression and suicidal thoughts. | ✖ |  |  |  | ✖ |  |  |  | Yes | ✖ |
| 23 | Client struggling with self-harm and intrusive thoughts. |  | ✖ |  |  | ✖ |  |  | ✖ | Yes |  |
| 24 | Client experiencing suicidality, anxiety; upset with friends and other social issues. | ✖ | ✖ |  |  |  |  | ✖ |  | Partial |  |
| 25 | Suicidal client in distress from job loss and dropping out of school. | ✖ |  |  |  |  |  | ✖ |  | Yes |  |
| 26 | Suicidal client struggling with racial profiling and issues at school. | ✖ |  | ✖ |  | ✖ |  |  | ✖ | No | ✖ |
| 27 | Client struggling with ongoing suicidal ideation, academic stress, and abuse at school. | ✖ |  |  |  |  |  |  | ✖ | No | ✖ |
| 28 | Client in distress; struggling with autism and health concerns. |  |  |  |  |  |  | ✖ | ✖ | No |  |
| 29 | Suicidal client struggling with relationship issues. | ✖ |  |  |  |  |  | ✖ |  | Yes |  |
| 30 | Peer seeking help for a suicidal classmate. | ✖ |  |  |  | ✖ |  |  |  | Yes | ✖ |
| 31 | Client experiencing family conflict, problems with social services, and depression. |  |  |  | ✖ |  | ✖ |  |  | Yes |  |
| 32 | Suicidal client struggling with active self-harm and academic stressors. | ✖ | ✖ |  |  |  |  | ✖ |  | Yes | ✖ |

^a^Non-suicidal self injury (NSSI) includes self harm without intent to die, such as cutting.

^b^Emergency triage includes triaging to emergency responders, hospital emergency rooms, mobile crisis outreach teams.

^c^Client drop off indicates the client stopped responding to the counselor.

^d^Resolve indicates reduction of client’s risk or distress and/or de-escalation of client crisis.

^e^Risk=Higher risk. Determination based on team assessment of encounters.

Table 6. Summary of False Positive encounters

|  |  | Post-hoc Human Rating | | | | | | | | | |
| --- | --- | --- | --- | --- | --- | --- | --- | --- | --- | --- | --- |
| ID | Encounter Summary | Suicidality | NSSI^a^ | Abuse | Emergency Triage^b^ | Mobile Tips | Social Services | External Therapy Services | Client Drop Off^c^ | Resolve^d^ | Risk^e^ |
| 1 | Client experiencing family conflict and emotional abuse. |  |  | ✖ |  |  |  |  |  | Yes |  |
| 2 | Client experiencing family conflict, emotional abuse, suicidal thoughts. | ✖ | ✖ | ✖ |  |  |  | ✖ |  | Partial |  |
| 3 | Parent concerned with child’s depression, suicidality; struggling with legal concerns. | ✖ |  | ✖ |  |  |  | ✖ | ✖ | Partial |  |
| 4 | Client experiencing active familial abuse. |  |  | ✖ |  |  |  |  | ✖ | No |  |
| 5 | Client struggling with rape and sexual assult; seeking resources. |  |  | ✖ | ✖ |  |  |  | ✖ | Partial |  |
| 6 | Client lost job and car; struggling with depression and suicidal thoughts. | ✖ |  |  | ✖ |  |  | ✖ |  | Partial | ✖ |
| 7 | Client struggling with perfectionism and self-harm as punishment. |  | ✖ |  |  |  |  | ✖ | ✖ | No |  |
| 8 | Suicidal client with a plan; struggling with family dynamics. | ✖ |  |  |  |  |  |  | ✖ | No | ✖ |
| 9 | Family abuse, rape, and recent suicide attempts. | ✖ | ✖ | ✖ |  | ✖ | ✖ |  | ✖ | Yes |  |
| 10 | Client struggling with loss of parent and other familial issues. |  |  |  |  |  |  |  | ✖ | Partial |  |
| 11 | Client struggling with illness, grief, and loss of family members. |  |  |  |  |  |  |  |  | Yes |  |
| 12 | Client struggling with self-harm and emotional triggers. |  | ✖ |  |  |  |  |  |  | Yes |  |
| 13 | Client experiencing suicidal thoughts; concerns with affording health care. | ✖ |  |  | ✖ |  |  | ✖ |  | Yes | ✖ |
| 14 | Peer seeking help for friend struggling with sexual assault and family conflict. |  |  | ✖ |  | ✖ |  |  | ✖ | No |  |
| 15 | Client distressed about anxiety, depression, and suicidality. | ✖ |  |  |  |  |  |  | ✖ | No | ✖ |
| 16 | Parent seeking resources for child who self-harmed. |  | ✖ |  |  |  |  | ✖ | ✖ | Partial |  |
| 17 | Client struggling with depression. |  |  |  |  |  |  |  |  | No |  |
| 18 | Peer seeking advice on how to help suicidal friend. | ✖ |  |  | ✖ |  |  |  |  | Yes |  |
| 19 | Suicidal client with a plan. | ✖ |  |  | ✖ |  |  | ✖ |  | Yes | ✖ |
| 20 | Client experiencing familial abuse, and rape; struggling with suicidal thoughts. | ✖ |  | ✖ | ✖ | ✖ | ✖ | ✖ |  | Partial | ✖ |
| 21 | Client struggling with suicidal thoughts; planning to overdose. | ✖ | ✖ |  |  |  |  |  |  | Yes | ✖ |
| 22 | Client upset with family response to past suicide attempt. | ✖ |  |  |  |  |  | ✖ |  | Yes |  |
| 23 | Client experiencing family abuse; and suicidality. | ✖ |  | ✖ | ✖ |  |  |  |  | Yes | ✖ |
| 24 | Client distressed by abusive ex; struggling with suicidal family. | ✖ |  | ✖ | ✖ |  |  |  | ✖ | Partial |  |
| 25 | Suicidal client struggling with active self-harm. | ✖ | ✖ |  |  |  |  | ✖ |  | Yes |  |
| 26 | Parent seeking help for suicidal, self- harming child; potential danger to others. | ✖ | ✖ |  | ✖ |  |  | ✖ |  | Yes | ✖ |
| 27 | Client upset by neglectful family; issues with access to healthcare. | ✖ |  |  |  |  |  | ✖ |  | Partial | ✖ |
| 28 | Client requesting SafeUT help the police locate them. |  |  |  | ✖ |  |  |  | ✖ | No |  |
| 29 | Parent seeking help for suicidal child with recent overdose; family conflict. | ✖ |  | ✖ | ✖ |  | ✖ | ✖ |  | Yes | ✖ |
| 30 | Client struggling with self-harm. |  | ✖ |  |  |  |  |  | ✖ | No | ✖ |
| 31 | Peer seeking help for suicidal friend; struggling with own suicidality. | ✖ |  |  |  | ✖ |  | ✖ |  | Yes |  |
| 32 | Client struggling with marital problems and depression. |  |  |  |  |  |  | ✖ | ✖ | Partial |  |
| 33 | Client struggling with suicidal thoughts; possible abuse of SafeUT services. | ✖ |  |  | ✖ |  |  | ✖ |  | Partial | ✖ |

^a^Non-suicidal self injury (NSSI) includes self harm without intent to die, such as cutting.

^b^Emergency triage includes triaging to emergency responders, hospital emergency rooms, mobile crisis outreach teams.

^c^Client drop off indicates the client stopped responding to the counselor.

^d^Resolve indicates reduction of client’s risk or distress and/or de-escalation of client crisis.

^e^Risk=Higher risk. Determination based on team assessment of encounters.
